# Clinical preceptor competencies for a better pharmacy education: a suggested framework for Lebanon

**DOI:** 10.1186/s40545-020-00217-3

**Published:** 2020-06-24

**Authors:** Abeer Zeitoun, Hala Sacre, Souheil Hallit, Rony M. Zeenny, Georges Sili, Pascale Salameh

**Affiliations:** 1 INSPECT-LB: Institut National de Santé Publique, d’Épidémiologie Clinique et de Toxicologie, Beirut, Lebanon; 2 Drug Information Center, Order of Pharmacists of Lebanon, Beirut, Lebanon; 3 Faculty of Medicine and Medical Sciences, Holy Spirit University of Kaslik (USEK), Jounieh, Lebanon; 4 Department of Clinical Pharmacy, American University of Beirut Medical Center, Beirut, Lebanon; 5 Continuing Education Department, Order of Pharmacists of Lebanon, Beirut, Lebanon; 6 Faculty of Medicine, Lebanese University, Beirut, Lebanon; 7 Faculty of Pharmacy, Lebanese University, Beirut, Lebanon

**Keywords:** Clinical preceptor, Competency framework, Experiential education, Lebanon

## Abstract

**Background:**

Experiential education is a core element in developing the ability of pharmacy students to apply theory to practice, solve problems, and acquire standard pharmacist competencies. To achieve this goal, preceptors play a crucial role as teachers, mentors, and evaluators. The study objective was to identify and describe the core competencies and skills considered essential to the success of clinical preceptor pharmacists in the training of future care providers, in a steadily changing environment.

**Methods:**

A task force was formed within the Scientific Committee of the Lebanese Order of Pharmacists to map, sort, select, and adapt international competencies to the current Lebanese context.

**Results:**

Seven roles were identified: Role model/Practitioner/Mentor, Supervisor/Facilitator/Coach/Teacher, Manager, Collaborator/Communicator, Leader, Clinical expert, and Researcher. Related competencies were also defined.

**Conclusion:**

all aforementioned competencies are expected to improve the acquisition of learning by pharmacy trainees in Lebanon. This framework can be used by pharmacy program directors and preceptors for developmental purposes through self- or peer-evaluation processes, identifying gaps, and helping to fill the needs for a better workforce in academic and experiential pharmacy education.

## Background

Experiential education is an essential element in developing the ability of pharmacy students to apply theory to practice, solve problems, and acquire standard pharmacist competencies. To achieve this goal, preceptors play a crucial role in the pharmacy practice experience, as teachers, mentors, and evaluators [1]. They guide students and help them engage in pharmacy activities and apply theoretical knowledge to actual clinical practice; they also evaluate their performance and provide useful feedback [2, 3].

In Lebanon, five universities teach pharmacy: two of them follow the French system, two others the American system, and one follows the Canadian system. Universities that follow the American system are required to have more than 30% of their pharmacy curricula in clinical practice settings. Indeed, to complete their Introductory Pharmacy Practice Experiences (IPPE), students are required to complete a minimum of 300 h in parallel with their theoretical courses. For those who wish to earn a Doctor of Pharmacy degree, this initial training is followed by a final year of full-time training of a minimum of 1440 h (Advanced Pharmacy Practice Experiences-APPE), as per the Accreditation Council for Pharmacy Education (ACPE) [4]. In the other educational systems, no minimum number of training hours is specified; however, the Lebanese system currently requires a minimum of 12 months of training in any pharmaceutical institution for any graduate wishing to practice pharmacy. Furthermore, two universities are currently offering postgraduate residency training for pharmacists [5].

Precepting trainees can be defined as “a practice of rendering a learner the chance to develop and utilize the superior skill and discipline of a profession in any clinical practice setting” [6]. It is an asset for the professional development of the pharmacy scholar and the future of the clinical pharmacy profession. It involves partnership, time investment, communication, tailoring of scholarly activities, team-work, coaching, evaluation of performance, role modeling, and counseling [6]. This practical experience allows learners to progress and shapes their values and attitudes [7, 8].

This supervised training aims at maturing the learner at both the professional and the personal level, thus leading to a better competence for practice after graduation. It is essential to continually assess the trainee throughout the period of clinical practice in order to identify difficulties and address them in a timely manner [6, 9].

Preceptors share their knowledge and experience with their students and residents and ensure that all supervised trainees will reach and develop the expected professional and clinical competencies necessary to practice at any pharmacy site [8]. To better prepare the students for their future clinical practice, clinical preceptors should build on the theoretical knowledge and skills acquired by students, which usually includes simulation scenarios and other active learning experiences. They should focus their teaching on three essential domains: professionalism, effective communication, and how to apply theoretical knowledge and skills earned from courses to real, dynamic patient care situations. To achieve these goals, preceptors must have a set of core values, technical skills, and abilities [6, 9]. They are required to hold a PharmD degree (or higher when precepting postgraduate pharmacy trainees) and to have practiced as pharmacists for a relevant period in the setting where precepting is to take place, hospital or community pharmacy, for example [6]. They should be available at the authorized training practice site on a regular basis or, while absent, appoint the supervision to a qualified adjunct pharmacist at the practice site [6].

In most countries, national boards are usually responsible for defining the legal responsibilities of preceptors, and schools of pharmacy set the educational requirements of clinical rotations, while defining clinical precepting skills and attributes differs between countries: in developed countries, several competencies frameworks were developed [10], while the issue remains vaguely identified in developing countries [11], particularly the Arab world. In Lebanon, the Lebanese Order of Pharmacists (OPL, the official pharmacists’ association in Lebanon) decided to work on this endeavor through the appointed scientific committee. The objective was to identify and describe the core competencies and skills deemed essential to the success of clinical preceptor pharmacists in today’s rapidly evolving healthcare environment.

## Methods

A core competency framework for pharmacists had been defined as a key requirement of the undergraduate and postgraduate education of pharmacists in Lebanon [12]. After developing the core competency framework for pharmacists, the OPL identified an urgent need to develop the minimal competencies needed for several pharmacy sectors/specializations, including those of clinical preceptor. A task force was formed within the OPL Scientific Committee to define these competencies, based on internationally available frameworks. Mapping, sorting, selecting, and adapting to the current Lebanese context led to sets of competencies categorized by domain, according to the required function/role of the preceptor.

## Results

Clinical preceptor competencies are reported in Table 1. Seven major roles, with details of behaviors and competencies to be fulfilled by role, were identified for the Lebanese clinical preceptor.

1. Role model/Practitioner/Mentor: The preceptor should try to develop at the professional level, seeking to acquire ethical and professional skills.
2. Supervisor/Facilitator/Coach/Teacher: The preceptor has a prominent role in supervising and teaching trainees and should have, therefore, effective and responsible clinical teaching skills. He should facilitate learning through coaching, listening, providing feedback, and applying active learning methods.
3. Manager: The preceptor should be able to manage all aspects of the training, including organizing activities, time management, and applying rules and regulations.
4. Collaborator/Communicator: The preceptor should have appropriate interpersonal and communication skills, in addition to cultural competency to effectively collaborate with trainees and other health professionals involved in the training.
5. Leader: The preceptor should have adequate leadership skills to motivate trainees and provide them with continuous development skills.
6. Clinical expert: The preceptor should have appropriate and up-to-date knowledge and clinical skills, in addition to other technical skills.
7. Researcher: The preceptor should engage in research projects and encourage students to present and publish their work; he should also be able to manage research.

## Discussion

Based on the identified roles and competencies, clinical preceptors in Lebanon should serve as a role model, implement professional values and attitudes, and usher trainees through the experiential education program, indispensable to complement their acquired academic knowledge and become competent pharmacists [6].

Clinical preceptors assume the role of the teacher during the clinical training period. The majority are university instructors, but some are practitioners on the training site and serve as adjunct preceptors; all have to teach in the uncontrolled environment of the clinical practice setting, adding to the complexity of the task [9]. They would function as a learning asset for trainees, guiding their learning by assessing their knowledge and clinical background experience at the beginning of their training experience. They would discuss their strengths and weaknesses, provide an orientation at the practice site, develop a generalized and individualized training plan, meet regularly with the trainees, review their progress, and discuss topics that can feed into developing required clinical skills. Clinical preceptors would also expose trainees to a full range of professional services, stimulating and challenging them to judge, acquire, and justify evidence-based decisions, and provide supportive and developmental feedback during the trainee learning experience. Unlike a classroom environment where the learning can be tailored to fit the learning process, the clinical practice has no borders of control, which can be very challenging for trainees. In these unacquainted surroundings, trainees look to the preceptor to seek guidance. This is why it is essential to set clear boundaries at the beginning of the training and reevaluate them as trainees progress in their experience where they would have gained confidence and acquired the required skills. Accordingly, the time and effort invested in orientation and practical education would translate into faster integration and a higher contribution of trainees to their workplace [6, 9].

Consequently, as the teaching occurs in an undetermined, modifiable, and original conditions, it is crucial to use a constant cognitive process of problem-solving and decision-making. It is also important to realize that what is taught is not necessarily the same as what the trainee acquires. Teaching should be a non-stop process of continuous interaction between the four elements of a learning experience: the teacher (preceptor), the learner (trainee), the subject, and the context [13].

Clinical preceptors have the responsibility to serve as a role model on how to support a professional development program. One of their duties is to lead the trainee by practical example, thus becoming a model and a mentor [13, 14]. Moreover, preceptors should direct trainees in some soft skills, including management, communication, collaboration, and leadership, and plan for increased inter-professional engagement during rotations [15, 16].

Finally, following the recommendations of the International Pharmaceutical Federation (FIP) and the World Health Organization (WHO), the pharmacist’s role should extend to research [15, 16]. In Lebanon, a study conducted by the OPL had revealed the readiness and willingness of Lebanese pharmacists to participate in research [17]. Therefore, preceptors should conduct and lead in research; all pharmacists, particularly those working in academia, should be interested in research to ensure their continuing education, update their knowledge, and generate new information to promote patient health. The modern pharmacist having major roles outside the clinical context [15, 16], the Lebanese pharmacist should be ready to become the “Nine Star Pharmacist”, with the appropriate professional attitude and ethical behaviors.

## Conclusion

In conclusion, this framework, suggested to pharmacy program directors and preceptors, can be used for developmental purposes through self- or peer-evaluation processes, identifying gaps, and helping to fill the needs for a better workforce in academic and experiential pharmacy education. All the competencies described are expected to improve the experiential learning of pharmacy in Lebanon.

## Supplementary information

**Additional file 1.**

## Data Availability

No data is available since this study did not involve any human participant.
