# Supplementary material for: Clinical preceptor competencies for a better pharmacy education: a suggested framework for Lebanon
Source: J Pharm Policy Pract. 2020 Jun 24;13:21. doi: 10.1186/s40545-020-00217-3 (PMC7313193; doi:10.1186/s40545-020-00217-3)
Supplement: Supplementary file 1 — Additional file 1. [file 40545_2020_217_MOESM1_ESM.pdf]

# Clinical Preceptor Minimal Competencies

| Functions | Domain | Competency | Behaviors |
|---|---|---|---|
| **Role model/ practitioner/mentor** | 0. Professional Development | 0.1. Ethical and professional skills | 0.1.1. Combine an empathetic and supportive attitude toward students while maintaining an unwavering commitment to excellence in patient care.<br>0.1.2. Combine a commitment to continuous quality improvement with reflection on action to support meaningful learning experiences.<br>0.1.3. Ensure that the pharmacy environment embodies the same principles of practice that they hope their student pharmacists will embrace.<br>0.1.4. Identify their own areas for improvement and be willing to openly discuss any issues or events that need improvement as a learning experience for both themselves and their students.<br>0.1.5. Demonstrate a caring attitude toward students, and patients<br>0.1.6. Discuss the rationale used in practice with students to make sure they understand specific behaviors<br>0.1.7. Commit to their organization, professional societies, and the community |
| **Supervisor/ Facilitator/Coach/ Teacher** | 1. Supervising and teaching skills | 1.1. Effective clinical teaching skills | 1.1.1. Assess learning opportunities for the students<br>1.1.2. Give students responsibilities for patient care while still monitoring their actions in a non-threatening way<br>1.1.3. Demonstrate procedures<br>1.1.4. Provide opportunities to do procedures<br>1.1.5. Observe student performance frequently<br>1.1.6. Correct students without belittling them<br>1.1.7. Provide supplementary learning activities, when needed, to achieve all expected learning opportunities |
| | | 1.2. Responsible teaching | 1.2.1 Are responsible for the actions of their students |
| | 2. Facilitating learning | 2.1. Coaching skills, listening skills, positive feedback | 2.1.1. Empower students to ask questions, search for answers, and become independent learners.<br>2.1.2. Question students thoughtfully and encourage feedback<br>2.1.3. Identify and respond to each student specific learning needs<br>2.1.4. Challenge the learning process in each student<br>2.1.5. Coach student behavior through effective and timely feedback<br>2.1.6. Prioritize students teaching as an important focus of the practice site |

DRAFT

| | | | |
|---|---|---|---|
| | | | 2.1.7. Treat students as colleagues in training |
| | | 2.2. Active learning facilitation | 2.2.1. Engage students in the process of active learning<br>2.2.2. Help students to become life-long learners<br>2.2.3. Encourage active participation in a group<br>2.2.4. Establish a harmonious relationship with students based on understanding and good communication<br>2.2.5. Show personal interest in students<br>2.2.6. Emphasize problem solving |
| **Manager** | 3. Clarity, organizational /time management skills and enthusiasm | 3.1. Organization Skills | 3.1.1. Explain clearly<br>3.1.2. Present material in an organized manner<br>3.1.3. Summarize information<br>3.1.4. Communicate what is expected to be learned<br>3.1.5. Teach by example<br>3.1.6. Answer questions carefully and precisely<br>3.1.7. Balance clinical and teaching responsibilities<br>3.1.8. Commit to continuous quality improvement for managerial techniques, clinical skills and practice site to support the professionalism of pharmacy practice |
| | | 3.2. Rules & Regulation | 3.2.1. Follow rules and regulations set by the program of experiential activities<br>3.2.2. Schedule and plan activities such as case discussions, journal club, topic presentations and other activities set by the program<br>3.2.3. Document and assess student performance |
| | | 3.2. Time management | 3.2.1. Manage their own time well, manage their student's time, and teach students to manage it efficiently.<br>3.2.2. Use tools that can help manage your time<br>3.2.3. Allocate activities through the rotation effectively.<br>3.2.4. Educate students about the role that time management plays in the financial health of any practice setting. |
| **Collaborator Communicator** | 4. Interpersonal/ Communication skills | 4.1. Communication Skills | 4.1.1. Interact and communicate effectively with administrators, healthcare colleagues, peers, patients, or students to reach desired outcomes.<br>4.1.2. Develop an effective student-preceptor relationship<br>4.1.3. Be willing to listen to the students and learn something new<br>4.1.4. Convey confidence in students' abilities to allow them to work harder to reach goals |

DRAFT

| | | | |
|---|---|---|---|
| | | | 4.1.5. Provide positive reinforcement and constructive feedback so that students can understand their strengths and improve their weaknesses<br>4.1.6. Possess conflict management skills, and be prepared to teach students to use them.<br>4.1.7. Use public speaking skills to speak effectively in large and small group situations.<br>4.1.8. Provide effective medication and onsite related education training/ presentations to students, pharmacists and other health care professionals. |
| | 5. Cultural competency skills | 5.1. Cultural competency skills | 5.1.1. Provide care to patients with diverse values, beliefs, and behaviors, including tailoring delivery to meet patients' social, cultural, and linguistic needs<br>5.1.2. Appreciate and value cultural differences to provide high-quality care to patients from diverse backgrounds.<br>5.1.3. Be prepared to respect and value these differences among students and collaborate in a manner that promotes education.<br>5.1.4. Strive to foster an attitude of cultural competence in their students<br>5.1.5. Encourage student pharmacists to accept and respect differences<br>5.1.6. Create an open and trusting environment for communication with people from diverse cultures<br>5.1.7. Be willing to self-assess their performance in working with patients from differing backgrounds.<br>5.1.8. Provide students with resources, such as links to Web sites that support cultural competency and diversity, to help expand their cultural knowledge |
| **Leader** | 6. Leadership skills | 6.1. Leadership skills | 6.1.1. Demonstrate effective managerial and leadership relationships with pharmacist colleagues and staff<br>6.1.2. Demonstrate aspects of humility related to one's own limitations<br>6.1.3. Monitor quality of professional practice and teaching activities<br>6.1.4. Evaluate and refine the practice and activities on an ongoing basis to continually improve their activities in the pursuit of excellence<br>6.1.5. Practice nondiscriminatory behaviors<br>6.1.6. Embody a personal practice philosophy<br>6.1.7. Demonstrate personal motivation and inspiring students and pharmacists to develop patient care practices |

DRAFT

| | | | |
|---|---|---|---|
| | | | 6.1.8. Have a mission or vision statement of pharmacy<br>6.1.9. Encourage administration to support patient care service |
| | | 6.2. Motivation | 6.2.1. Have the desire to educate others (patients, care givers, other health care professionals, students, pharmacy residents)<br>6.2.2. Demonstrate enjoyment and enthusiasm for patient care and teaching<br>6.2.3. Adapt an interesting style of presentation to capture student's attention<br>6.2.4. Stimulate interest in the subject and motivate students to learn |
| | | 6.3. Continuous Development skills | 6.3.1. Be active in professional organizations<br>6.3.2. Have a systematic, self-directed approach to their own continuing professional development |
| **Clinical Expert** | 7. Knowledge and clinical skills | 7.1. Clinical Skills | 7.1.1. Possess clinical skills and use them in practice |
| | | 7.2. Other technical skills | 7.2.1. Use effective educational techniques in the design of all educational activities.<br>7.2.2. Use information technology to make decisions and reduce error. |
| **Researcher** | 8. Research and project management skills | 8.1. Research and project management skills | 8.1.1. Participate in a practice-related research project using effective project management skills<br>8.1.2. Encourage students to present and publish their work on projects |

DRAFT
